# Immunotherapy targeting the PD-1 pathway alleviates neuroinflammation caused by chronic *Toxoplasma* infection

**DOI:** 10.1038/s41598-023-28322-8

**Published:** 2023-01-23

**Authors:** Jianchun Xiao, Ye Li, Treva Rowley, Jing Huang, Robert H. Yolken, Raphael P. Viscidi

**Affiliations:** 1 Stanley Division of Developmental Neurovirology, Department of Pediatrics, Johns Hopkins School of Medicine, Baltimore, MD 21287 USA; 2 Department of Pediatrics, Johns Hopkins School of Medicine, Baltimore, MD 21287 USA

**Keywords:** Microbiology, Neuroscience

## Abstract

*Toxoplasma gondii* can infect the host brain and trigger neuroinflammation. Such neuroinflammation might persist for years if the infection is not resolved, resulting in harmful outcomes for the brain. We have previously demonstrated the efficacy of immunotherapy targeting the programmed cell death protein 1 (PD-1) pathway on clearance of *Toxoplasma* tissue cysts. We aimed to test whether parasite clearance would lead to the resolution of neuroinflammation in infected brains. We established chronic *Toxoplasma* infection in BALB/c mice using the cyst-forming Prugniaud strain. Mice then received αPD-L1 or isotype control antibodies. After completion of the therapy, mice were euthanized six weeks later. The number of brain tissue cysts, *Toxoplasma*-specific CD8 + T cell proliferation and IFN-γ secretion, serum cytokine and chemokine levels, and CNS inflammation were measured. In αPD-L1-treated mice, we observed reduced brain tissue cysts, increased spleen weight, elevated IFN-γ production by antigen-specific CD8 + T cells, and a general increase in multiple serum cytokines and chemokines. Importantly, αPD-L1-treated mice displayed attenuation of meningeal lymphocytes, reactive astrocytes, and C1q expression. The reduction in inflammation-related proteins is correlated with reduced parasite burden. These results suggest that promoting systemic immunity results in parasite clearance, which in turn alleviates neuroinflammation. Our study may have implications for some brain infections where neuroinflammation is a critical component.

## Introduction

*Toxoplasma gondii* can infect the brain and trigger neuroinflammation. Inflammatory response includes activation of microglia and astrocyte, upregulation of complement components, infiltration of immune cells, and elevation of cytokines and chemokines^1–4^. Although these inflammatory processes effectively control parasite reactivation, the state of neuroinflammation may persist for years if the infection does not resolve. Persistent inflammation has been associated with multiple neuropathologies in *Toxoplasma*-infected animals. Studies have reported cortical neurodegeneration^5^, changes in neuronal morphology^6^, brain connectivity^7^, and neurotransmitter pathways^8^. Neuroinflammation is a critical component of virtually all neurodegenerative diseases. Not surprisingly, *Toxoplasma* seroprevalence in humans has been considered a risk factor for various brain disorders such as schizophrenia, Alzheimer’s, and Parkinson’s disease^9^.

*Toxoplasma* tissue cysts are reported to significantly affect inflammatory responses^1,5,10–12^. As revealed by transcriptome analysis of host brains, expression of many immune- and neuronal-related genes was mainly associated with cyst burden. Tanaka et al.^10^ found positive correlations between the number of parasites in the infected mouse brains and the expression levels of genes involved in host immune responses. In contrast, genes that had a negative correlation with parasite numbers were those genes that are predicted to be involved in neurological functions, such as small-GTPase-mediated signal transduction and vesicle-mediated transport. Boillat et al.^11^ observed a link between the number of tissue cysts and markers associated with pro-and anti-inflammatory cascades, astrocyte activation, neuronal loss, and downregulation of neurotransmitter pathways. The plasma level of proinflammatory cytokines, such as IFN-γ and IL-12/IL-23p40, have also been associated with cyst burden. We have observed that parasite burden is a critical determinant for expressions of several inflammatory molecules (CCL5, IL-12p70, IL-12p40, and TNF-α) and markers for synaptic remodeling (C1q) and neuronal cell damage (FJB)^1,5,12^. Such changes were not present in mice with lower cyst burden or mice that did not progress to the chronic stage of infections (cyst free).

Neuroinflammation is defined as an inflammatory response within the brain or spinal cord, with signals originating from barrier tissues such as brain vasculature, surrounding meninges, and the choroid plexus^13–15^. The meninges enclose the brain and spinal cord, which have traditionally been considered structures that protect the brain parenchyma. However, recent studies suggest that the meninges are an immunologically active compartment communicating with the periphery via the meningeal lymphatic system^16^. Increasingly studies demonstrate that meninges play a critical role in the maintenance of brain function and CNS disease^17–19^. Walker-Caulfield et al.^20^ reported that meningeal inflammation precedes inflammation in the CNS and parallels remittances and relapses in a murine model of multiple sclerosis.

Studies have shown that diminishing the inflammatory responses rescues *Toxoplasma*-associated neuropathogenesis and behavioral abnormalities^21,22^. Although the treatments used in these studies may not eliminate tissue cysts, they significantly reduced the expression of inflammatory mediators in the brains of infected mice. As described above, cyst burden is a primary determinant of neuroinflammation. We hypothesized that diminishing neuroinflammation by clearance of the parasite is superior to anti-inflammatory drugs. Currently, available anti-*Toxoplasma* drugs are ineffective against tissue cysts. We have previously demonstrated that blockade of the programmed cell death protein 1 (PD-1) pathway significantly reduces the number of *Toxoplasma* tissue cysts in the brain^23^. The effect was associated with increased immune cell infiltration into the CSF-filled compartments (ventricles and subarachnoid space)^23^. Chronic *Toxoplasma* infection can result in CD8 + T-cell exhaustion^24^, a state in which these cells lose their function^25^. Exhausted T cells also increase the expression of inhibitory receptors, including PD-1, which prevent the activation of T-cells. The rationale of anti-PD-1 immunotherapy is that blocking PD-1 or its ligand PD-L1 restores T cell function^25^.

We tested whether parasite clearance via immunotherapy targeting the PD-1 pathway would attenuate brain inflammation. We previously used an outbred CD-1 mouse model of *Toxoplasma* infection with a virulent strain^23^. This model resembles the heterogeneous effects of *Toxoplasma* infection in humans. However, the model generates a limited number of tissue cysts (~ 200) because virulent strains do not readily develop tissue cysts in these mice. A limitation is the lack of well-defined T-cell epitopes elicited by *Toxoplasma* in CD-1 mice, making the enumeration of antigen-specific T-cell responses difficult. Here we used inbred BALB /c mice susceptible to a cyst-competent *Toxoplasma* strain and amenable to immunological studies. Specific objectives included: (1) will the treatment apply to a different model of *Toxoplasma* infection? (2) will the treatment reverse the phenotype of exhausted antigen-specific CD8 + T cells? (3) will the treatment reduce neuroinflammation?

## Methods

### Mouse model of chronic *Toxoplasma* infection

The protocol was approved by the Animal Care and Use Committee at Johns Hopkins University. All experiments were performed in accordance with the U.S. National Institutes of Health Guide for the Care and Use of Laboratory Animals, and all methods are reported in accordance with ARRIVE guidelines.

We established chronic infection of BALB/c mice with *Toxoplasma* Prugniaud (Pru) strain. BALB/c mice were chosen because three *Toxoplasma*–derived CD8 + T cell epitopes mediating L^d^-restricted protective immunity have been identified in the BALB/c strain^26,27^. The Pru strain is cyst-competent^28^. Briefly, 9-week-old male BALB/c mice (The Jackson Laboratory) were infected intraperitoneally (i.p.) with 400 tachyzoites of Pru strain. Control mice received vehicle only (phosphate-buffered saline [PBS]). Male mice were infected because our previous study showed males generate more tissue cysts than females^29^.

Infection was confirmed serologically by the presence of IgG antibodies to the whole *Toxoplasma* organism and peptide antigens of the *Toxoplasma* cyst protein MAG1. The anti-*Toxoplasma* antibodies were measured using a modified commercial ELISA kit^12^, and anti-MAG1 antibodies were determined using a previously developed MAG1 ELISA assay^30^. Serum was diluted at 1:100 for antibody testing.

### Mice grouping

At six weeks post-infection (wpi) when the maximum number of tissue cysts is supposed to reach the brain, blood was collected from the tail vein of the mice, and sera were isolated. *Toxoplasma* IgG and MAG1 antibodies were measured. Mice were then assigned to three groups, infected (n = 5), αPD-L1 (n = 7), and isotype control (n = 5), stratified by serum MAG1 level. MAG1 antibody is a serological marker for brain tissue cysts^12^, and the level of antibody varies by the number of cysts in the brain. The stratification ensured cyst burden would not differ in the three groups before treatment. The group of mice that were mock-infected with PBS (n = 5) served as uninfected controls.

### Reagents

Antigen and peptide selection. Three *Toxoplasma*–derived L^d^-restricted CD8 + T cell epitopes, the GRA6-derived HF10 (HPGSVNEFDF)^26^, the ROP7-derived IF9 (IPAAAGRFF), and the GRA4-derived SM9 (SPMNGGYYM) peptides^27^, were synthesized by GenScript with high purity (> 95%).

H-2L^d^ HF10 (HPGSVNEFDF) tetramers for detection of GRA6-specific CD8 cells were obtained from the NIH Tetramer Core Facility.

### In vivo blockade of the PD-1 pathway

Anti-PD-L1 antibodies were administrated to infected mice at 6 wpi. For blockade of the PD-1 pathway, mice received via i.p. 200 μg of rat antimouse PD-L1 antibody (10F.9G2, Biolegend) every 3 days for 2 weeks. Mice that received IgG2b antibody served as isotype control. The ability of this anti-PD-L1 antibody regimen to block the PD-1 pathway has been previously demonstrated^23^. Mice were sacrificed six weeks following the final injection, and brains, spleens, and blood were collected.

### Cell preparation and flow cytometry

Spleen cells from mice were collected by splenic grinding, each step with 5 mL RPMI‐1640 (Gibco). Cells were subsequently washed twice and then resuspended in RPMI‐1640. For peptide stimulation, cells were incubated overnight with individual peptides or a mixture of the 3 peptides in the presence of Brefeldin A. Activation cocktail (Biolegend) was used as a positive control. Background stimulation was assessed by stimulation with PBS control. Antigen-specific CD8 + T cells were analyzed by staining for CD3 (clone 17A2), CD8 (clone 53–6.7), and intracellular IFN-γ (clone XMG1.2) using a flow cytometer in the Johns Hopkins flow cytometry core. Data were acquired and analyzed using BD FACSDiva™ software.

### Serum cytokine quantification

When mice were sacrificed, blood was collected and serum isolated. Levels of 31 cytokines and chemokines in the serum were measured using Bio-Plex multiplex assay (Bio-Rad) including: BCA-1/CXCL13, IL-4, MIP-1α /CCL3, CTACK/CCL27, IL-6, MIP-1β/CCL4, ENA-78/CXCL5, IL-10, MIP-3α/CCL20, Eotaxin/CCL11, IL-16, RANTES/CCL5, Eotaxin-2/CCL24, IP-10/CXCL10, MIP-3β/CCL19, Fractalkine/CX3CL1, I-TAC/CXCL11, SCYB16/CXCL16, GM-CSF, KC/CXCL1, SDF-1α/CXCL12, I-309/CCL1, MCP-1/CCL2, TARC/CCL17, IFN-γ, MCP-3/CCL7, TNF-α, IL-1β, MCP-5/CCL12, IL-2, MDC/CCL22. All samples were analyzed according to the manufacturer’s instructions.

### Cyst enumeration

The brain was cut sagittally along the midline. One-half of the brain was used for the preparation of homogenates, while the other half served for immunohistochemical analyses. Tissue cyst was enumerated in brain homogenates as described previously^12^. Briefly, samples were examined using a fluorescent microscope, and the number of brain cysts was counted in seven samples of 8 μl suspension per brain homogenate. All numbers reported correspond to the numbers obtained for the half-brain multiplied by 2.

### Meninges collection

Meninges were collected as described by Louveau et al.^31^. Briefly, the skull was isolated, and the inferior jaw, lower orbits, and nasal bone were removed. The skullcap was obtained by cutting the post-tympanic hook and placing it onto a petri dish with ice-cold PBS. Under a dissecting binocular, the meninges were harvested with forceps starting at the level of the olfactory lobe. The tissues were then snap-frozen.

### Staining

The whole meninges were mounted and stained with DAPI (4′,6-diamidino-2-phenylindole; Sigma-Aldrich), rat anti-mouse anti-CD3 antibody (monoclonal, eBioscience, cat. 14-0032-85, dilution 1:1000), and anti-mouse anti-CD14 antibody (monoclonal, cat. 11-0141-82, 1:50, eBioscience). Images were visualized using a confocal laser microscope (Zeiss LSM700).

### Immunoblot analyses

Total protein was extracted from brain homogenates using T-PER™ Tissue Protein Extraction Reagent (Thermo Scientific) added with Halt Protease and Phosphatase Inhibitor Cocktail (Thermo Scientific). Protein concentrations were measured using a BCA protein assay kit (Thermo Scientific). Total protein (10 ~ 40 μg) was loaded on 4–20% TGX protein gel (Bio-Rad) for electrophoresis under non-reducing or reducing conditions and transferred to PVDF membranes (Bio-Rad). The membranes were blocked with Starting Block T20 (TBS) Blocking Buffer (Thermo Scientific) for 1 h at room temperature, followed by incubation with primary antibodies at 4 °C overnight. Proteins were probed with primary antibodies for IBA1 (polyclonal, cat. 016-20001, 1:400, Wako), GFAP (polyclonal, cat. ab7260, 1:500, Abcam), C1q (monoclonal, cat. ab71089, 1:1000, Abcam), and C4 (monoclonal, cat. NB200-541, 1:10, Novus Biologicals). Bands were visualized using enhanced chemiluminescence (SuperSignal West Femto Maximum Sensitivity Substrate, Thermo Scientific). Protein values were normalized for corresponding values of β-actin. Relative optical density was determined using ImageLab software (Bio-Rad).

### Statistical analysis

The significant differences between the αPD-L1 and isotype control groups were analyzed by Student’s t-test. For cytokine analysis, we then performed multiple testing corrections with a false discovery rate (FDR) set at 5%, in addition to a 1.5-fold change threshold, to detect upregulated molecules between the two groups. For multiple groups, ANOVA with Bonferroni's multiple comparisons was applied. Paired t-test was used to compare the mean ODs of MAG1 antibodies before and after treatment. Correlation analysis was performed using Pearson’s correlation coefficient (r). Data are presented as means ± SEM. Statistical analyses were conducted in Graph-Pad Prism V9.2.0. Significance was denoted as a *P* of < 0.05.

## Results

### *Toxoplasma* infection generates a variable number of tissue cysts in the brains of BALB/c mice

There were high levels of *Toxoplasma* IgG antibody in all exposed mice (OD = 4.0 ± 0). However, levels of MAG1 antibody varied greatly among these mice (OD = 0.88 ± 0.11), suggesting individual mice have different parasite burdens. In our previous studies, parasite burden was correlated with MAG1 antibody levels^12,29,30^. MAG1 is a protein abundantly expressed in the cyst wall surrounding the bradyzoites and within the cyst^32^. Because the initial parasite burden might affect the efficacy of immunotherapy, we stratified mice before treatment to have comparable levels of tissue cysts for each treatment group. Mice were assigned to three groups, and MAG1 levels in the infected group ranged from 0.30 to 1.16 (mean OD = 0.80 ± 0.15), in the αPD-L1 group from 0.44 to 1.26 (OD = 0.81 ± 0.12), and in the isotype control from 0.39 to 1.51 (OD = 0.86 ± 0.20). There were no significant differences in MAG1 levels among the three groups (*p* = 0.91).

### PD-L1 blockade effectively reduces brain tissue cysts

**Figure 1:**
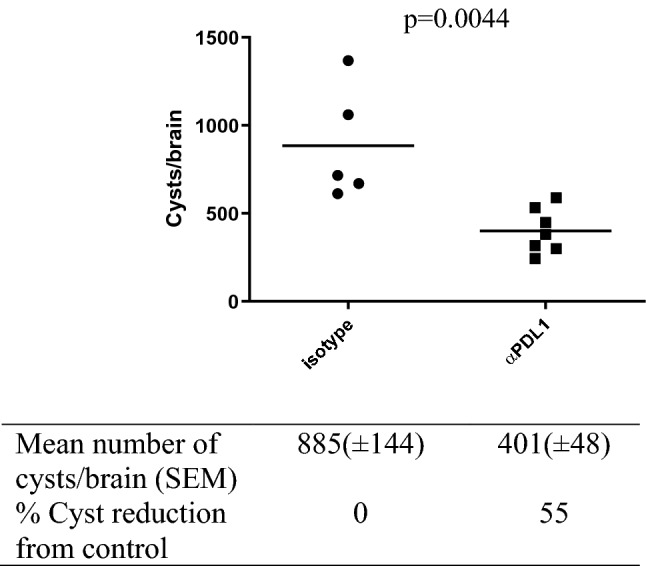
The efficacy of αPD-L1 monoclonal antibodies against latent *Toxoplasma* infection. Nine-week-old BALB/c mice were infected i.p. with 400 tachyzoites of Pru strain. At 6 wpi, anti-PD-L1 antibodies or isotype controls were administrated to infected mice every 3 days for 2 weeks. Six weeks after the final injection, mice were sacrificed to count the number of tissue cysts in the brain. Compared to isotype control, the number of tissue cysts was reduced by 55% in mice treated with αPD-L1. *P* value is calculated by Student’s t-test. Shown are the individual data points with a mean line.

In our previous study^23^, we show tissue cyst reduction occurs two weeks following the completion of the PD-L1 injection. Here, the mice were euthanized six weeks post-treatment because we hypothesized that neuroinflammation resolution would be a slower process than parasite clearance. We found a significantly lower number of brain tissue cysts in αPD-L1–treated mice than in isotype–treated (mean: 401 vs. 885, *p* = 0.0044, Fig. 1), demonstrating the therapeutic efficacy of the PD-L1 blockade in parasite control (55% reduction). To determine if reactivation from bradyzoite to tachyzoite occurs, we measured the tachyzoite-specific SAG1 gene. No SAG1 gene expression was detected in the brain tissues of both groups (data not shown).

### PD-L1 blockade leads to heavy spleen

**Figure 2:**
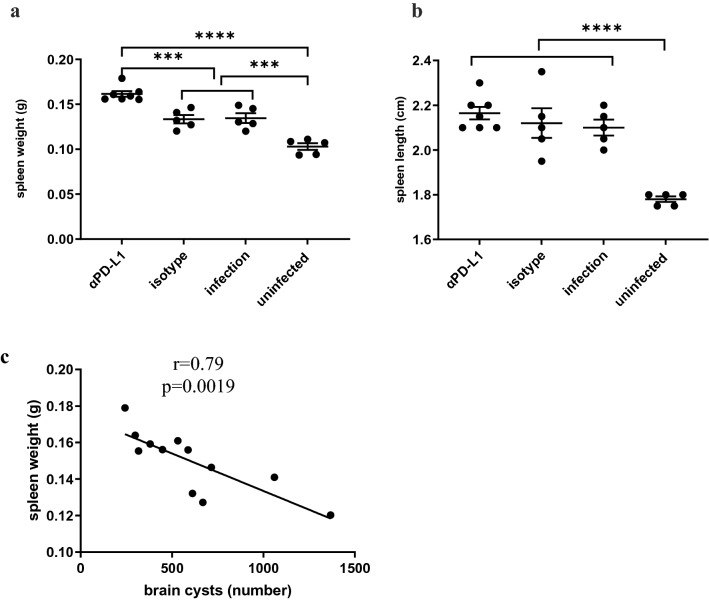
Effects of anti-PD-L1 treatment on mouse spleens. Differences in spleen weights (**a**) and lengths (**b**) were shown among anti-PD-L1-treated, isotype-treated, infected, and uninfected mice. (**c**) A negative correlation between the number of brain tissue cysts and spleen weight in *Toxoplasma*-infected mice treated with either PD-L1 or isotype control (n = 12). Statistically significant differences were determined by one-way ANOVA followed by Bonferroni’s correction for multiple comparisons (**a** & **b**) or Pearson’s correlation analysis (**c**). Data are shown as mean ± SEM, with individual data points. ***, *P* < 0.001; ****, *P* < 0.0001.

The spleen is a major immune organ playing a critical role in innate and adaptive immune responses. At the time mice were euthanized, we measured spleen weight and size. We found that the spleen became heavy after infection (mean weights of infected 0.135 g and uninfected 0.103 g; *p* = 0.0004, Fig. 2a). This result is consistent with previous studies^33,34^. Interestingly, the spleen became even heavier after the aPD-L1 blockade (0.162 g, Fig. 2a), and the differences were significant as compared to infected (0.137 g, *p* = 0.0021) and isotype-treated mice (0.133 g, *P* = 0.0008). We also found that the spleen became longer after infection (mean lengths of infected 2.117 cm and uninfected 1.780 cm; *p* < 0.0001, Fig. 2b). However, there was no further effect of the αPD-L1 blockade on spleen length as compared to infected mice and isotype-treated mice (2.164 cm, 2.117 cm, 2.120 cm, respectively; *P*s > 0.99, Fig. 2b). As expected, infected and isotype-treated mice had comparable spleen weight and length (*P*s > 0.99).

The number of brain cysts enumerated at the end of the experiment across both αPD-L1 and isotype control groups was assessed with spleen weight/length. We observed a negative correlation between the number of tissue cysts and spleen weight in these mice (r =  − 0.85, *p* = 0.0008, Fig. 2c), indicating the lower the spleen weight, the higher the cyst burden in this setting. There was no significant correlation between spleen length and cyst burden (*p* = 0.197).

### PD-L1 blockade promotes antigen-specific CD8 + T cell secretion of IFN-γ

**Figure. 3:**
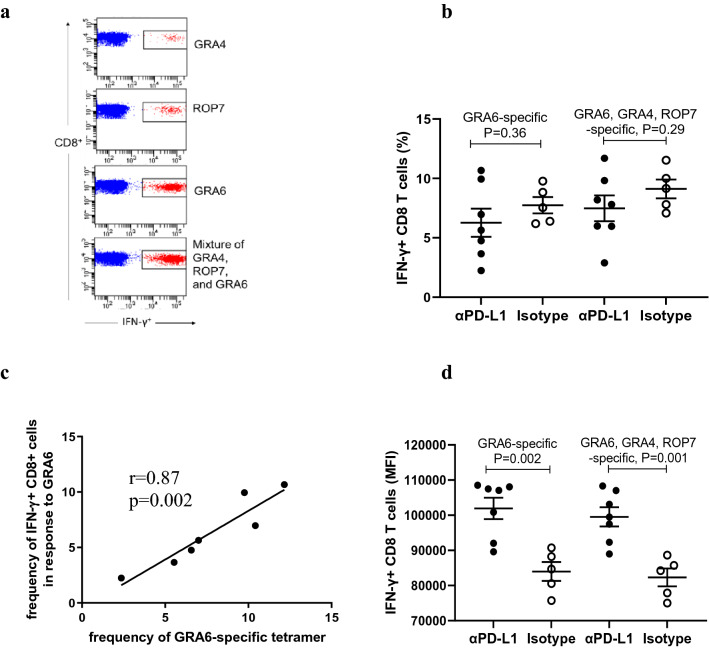
Frequency of antigen-specific CD8 + T cell response and intensity of IFN-γ staining after PD-L1 blockade. Splenocytes were harvested from *Toxoplasma*-infected mice treated with αPD-L1 (n = 7) or isotype control (n = 5). Cells were incubated overnight with individual peptides (GRA6, GRA4, and ROP7) or a mixture of the 3 peptides, followed by CD3, CD8, and IFN-γ staining. (**a**) Representative flow cytometry staining of GRA6, GRA4, ROP7, and pooled peptides-specific IFN-γ-secreting CD8 + T cells. (**b**) The frequency of GRA6-specific and pooled peptide-specific IFN-γ-secreting CD8 + T cells of αPD-L1 and isotype-treated mice. (**c**) Correlation between the frequency of GRA6 tetramer-positive CD8 + T cells and GRA6-specific IFN-γ-secreting CD8 + T cells. (**d**) The mean fluorescence intensity (MFI) of IFN-γ staining of CD8 + T cells specific for GRA6-epitope or the mixture of the 3 epitope-specific peptides in αPD-L1 and isotype-treated mice. Data are shown as mean ± SEM, with individual data points. P values are calculated by Student’s t-test (**b** & **d**) or Pearson’s correlation analysis (**c**).

CD8 + T cells and IFN-γ are significant effectors that mediate resistance to *Toxoplasma* infection^35^. We investigated whether antigen-specific CD8 + T cells are dysfunctional in IFN-γ production and proliferation upon antigen re-stimulation. Previous work has identified MHC class I-restricted epitopes in three *Toxoplasma* proteins (GRA6, ROP7, and GRA4) in the genetic background of BALB/c mice^26,27^. We determined the frequency of epitope-specific CD8 + T cells in splenocytes of *Toxoplasma*-infected mice following stimulation with individual peptides overnight. The frequency of IFN-γ secreting antigen-specific CD8 + T cells was 6.07%, 1.14%, and 0.36% for GRA6-, ROP7-, and GRA4-specific epitopes (Fig. 3a). Our results suggested that GRA6-specific CD8 + T cells are immunodominant, consistent with previous studies^26^.

We then examined the effect of PD-L1 blockade on GRA6-specific CD8 + T cell expansion and IFN-γ production. After overnight stimulation with GRA6 peptide, αPD-L1-treated mice had a frequency of IFN-γ secreting GRA6-specific CD8 + T cells of 6.27% compared to 7.75% for isotype-treated mice. The difference between PD-L1 and isotype groups was not significant (*p* = 0.3588, Fig. 3b). We also measured the frequency of total epitope-specific CD8 + cells by stimulating splenocytes with a peptide pool consisting of the three peptides. The frequency of IFN-γ secreting antigen-specific CD8 + T cells was 7.48% and 9.11% in mice treated with aPD-L1 and isotype control, respectively. Similarly, the difference between PD-L1 and isotype controls groups was non-significant (*p* = 0.2889, Fig. 3b). Our results show that PD-L1 blockade did not increase the frequency of IFN-γ producing antigen-specific CD8 + T cells in the spleen.

As another measure of antigen-specific CD8 + T cell response, we enumerated GRA6 tetramer + CD8 + cells. Splenocytes were stained with tetramers specific to the GRA6 epitope. The frequencies of tetramer-positive CD8 + T cells were 7.69% in mice with αPD-L1, while the frequencies were 10.00% in mice with isotype control. The difference between the two groups was not significant (*p* = 0.2076), confirming there was no GRA6-specific T-cells expansion. As expected, there was a strong positive correlation between the frequencies of GRA6- tetramer-positive CD8 + T cells and IFN-γ-secreting GRA6-specific CD8 + T cells in αPD-L1-treated mice (r = 0.87, *p* = 0.0022, Fig. 3c).

We then measured the fluorescence intensity of IFN-γ staining of antigen-specific CD8 + T cells. As shown in Fig. 3d, blockade with αPD-L1, as compared to isotype control, induced an increase in IFN-γ production by GRA6-specific CD8 + T cells (mean fluorescence intensity (MFI), 101,937 vs. 83,984, *p* = 0.0018) and similarly for CD8 + T cells stimulated with the pool of three peptides (MFI, 99,530 vs. 82,301, *p* = 0.0012,). These results demonstrate that antigen-specific CD8 + T cells produced more IFN-γ in mice treated with αPD-L1 than in isotype control, suggesting that PD-L1 blockade rescues an exhausted CD8 + T cell response.

### PD-L1 blockade induces a generalized increase in serum cytokine and chemokine levels

We investigated whether PD-L1 blockade affects the serum cytokine and chemokine levels. Employing Bio-Plex multiplex assay, we quantified 31 serum cytokines and chemokines. An increase in all cytokines and chemokines except CXCL16 was observed in αPD-L1-treated mice (Supplementary Excel spreadsheet 1). Using the cut-off criteria for multiple comparisons described in Statistical analysis (5% FDR, 1.5 fold change), we found that 13 of 30 cytokines and chemokines were significantly elevated. The increased expression occurred in both pro- and anti-inflammatory cytokines involving IFN-γ, IL-1β, IL-2, IL-4, IL-10, IL-16, and TNF-α. The increased expression of chemokines included the T cell chemoattractants CCL5, CXCL11, CCL27 and several leukocytes chemoattractant CX3CL1, CCL20, and CCL22. Among these molecules, CCL27 and IL-10 showed the highest fold change, 41 and 25-fold, respectively.

### PD-L1 blockade diminishes meningeal lymphocytes

**Figure 4:**
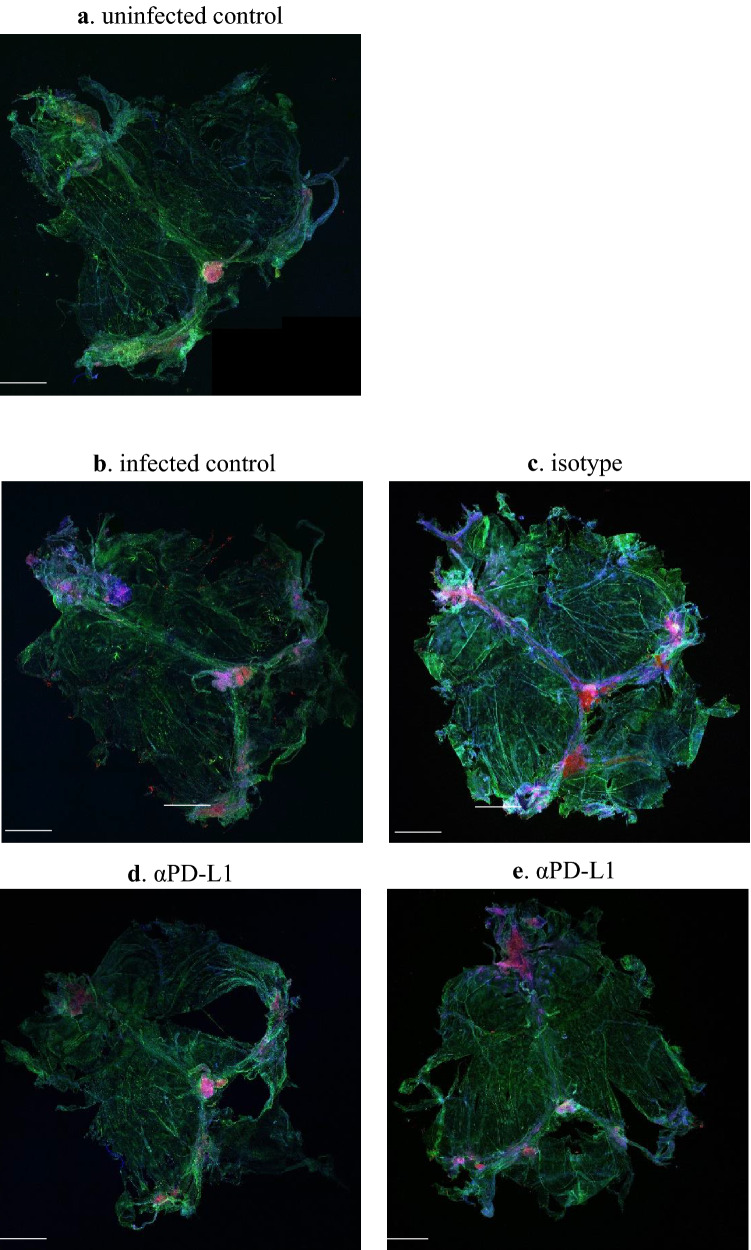
Leukocytes diminished in the meninges 6 weeks after αPD-L1 treatment. Representative images of CD3e (red), CD14 (green) labelling in whole-mount meninges (scale bar, 2000 mm) for uninfected control (**a**), infected control (**b**), isotype (**c**), αPD-L1 (**d** & **e**). DAPI was used to identify nuclei (blue).

The meningeal compartment is a direct route for immune-related communication between the central nervous system and the peripheral immune system^19^. The whole mount of mouse brain meninges was dissected and stained for CD3e (T cells), CD14 (monocytes), and DAPI (nuclei). Labeling of these cells in uninfected mice revealed a baseline level of expression (Fig. 4a). *Toxoplasma* infection induced an accumulation of CD3 + T lymphocytes in the meninges, with a high concentration of cells found close to the dural sinuses, as shown in both infected and isotype-treated mice (Fig. 4b,c). Moreover, CD14 staining was more pronounced throughout the meningeal compartments in infected and isotype-treated mice (Fig. 4b,c). Interestingly, the staining intensity of CD3 and CD14 was decreased in αPD-L1-treated mice (Fig. 4d,e). These results suggested that T lymphocytes and monocytes were reduced in the meninges six weeks after the completion of PD-L1 treatment.

### PD-L1 blockade suppresses astrocyte and complement activation

**Figure 5:**
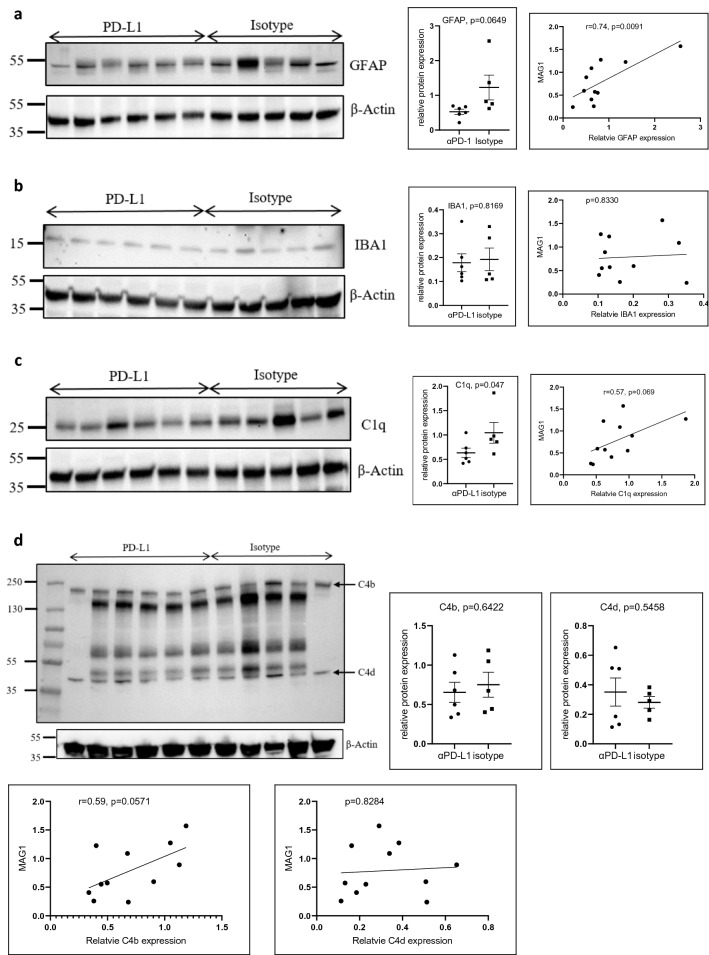
PD-L1 blockade on the suppression of glial and complement activation. Protein expression levels of GFAP (**a**), IBA1 (**b**), C1q (**c**), and C4 (**d**) were compared in brain homogenates of mice that received αPD-L1 (n = 6) and isotype control (n = 5), alongside β-actin loading controls by western blot. The analysis was run on a 4–20% polyacrylamide gel under either reducing (**a**, **b**, **c**) or non-reducing conditions (**d**). Histograms indicate densitometric analysis of blots, expressed as means + SEM, with individual data points. The relative protein expression was assessed with MAG1 antibody levels measured post-treatment by Pearson’s correlation analysis (n = 11). Analysis was performed in three independent experiments. The dots show individual values from one representative experiment. Significance was determined with a student's t-test.

We examined the extent of the brain inflammatory response since meningeal lymphatic vessels communicate with the brain parenchyma^16^. The primary CNS-resident cells involved in inflammation are astrocytes and microglia. GFAP and IBA-1 are well-recognized markers of activated astrocytes and microglia, respectively. We previously identified the activation of cerebral complement components in chronic *Toxoplasma* infection^1,5^. We thus compared the expression of these markers in brain homogenates of mice that received αPD-L1 or isotype control. Western blots showed that the expression of GFAP and C1q in αPD-L1-treated mice was lower than that in isotype-treated mice (GFAP, mean: 0.527 vs. 1.227, *p* = 0.0649; C1q, mean: 0.633 vs. 1.045, *p* = 0.047; Fig. 5a–c). However, the expression of IBA1 and C4b/d did not differ between the two groups (*P*s > 0.5, Fig. 5b,d). Under the non-reducing condition, immunoblot with anti-C4b/d antibody revealed an approximately 200 kDa band corresponding to the molecular weight of mouse C4b and a ~ 45 kDa band corresponding to the molecular weight of C4d (Fig. 5d). MAG1 antibody levels measured post-treatment correlated with the levels of GFAP (r = 0.74, *p* = 0.0091, Fig. 5a), and a trend towards significant correlation with C1q (r = 0.57, *p* = 0.069, Fig. 5c) and C4b (r = 0.59, *p* = 0.0571, Fig. 5d).

### Cyst reduction affects the MAG1 antibody level

**Figure 6:**
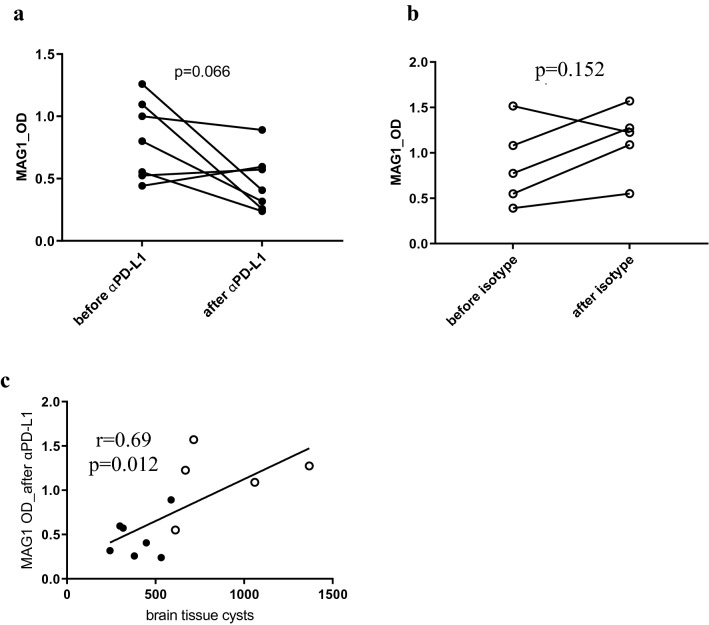
Changes in MAG1 antibody levels before and after the αPD-L1 or isotype treatment. Comparison of the MAG1 antibodies pre- and post-treatment for αPD-L1 (**a**) (n = 7, *p* = 0.066) and isotype (**b**) (n = 5, *p* = 0.153) mice. The number of tissue cysts enumerated in the brains of αPD-L1-treated and isotype control mice at the end of therapy correlated with MAG1 antibody levels measured post-treatment (**c**). *P* values are calculated by paired t-test (**a** & **b**) or Pearson’s correlation analysis (**c**). Black dot: αPD-L1 mice; circle: isotype mice.

At the start of treatment, the αPD-L1 and isotype control mice by design had comparable levels of MAG1 antibody, as noted above. Since the cyst burden at the end of treatment was lower in αPD-L1-treated mice than in isotype control mice, we determined whether the antibody levels changed over the treatment. As shown in Fig. 6a, MAG1 antibody levels decreased six weeks following the PD-L1 treatment (mean OD from 0.81 to 0.46), although the changes were highly variable, from negative to a decrease of 0.854 OD units. The decline showed a trend toward significance (Paired t-test, *p* = 0.0662). In contrast, MAG1 antibody levels showed a general increase in the isotype control group (mean OD from 0.86 to 1.14, Fig. 6b), although not significant (*p* = 0.1518). A correlation between change in MAG1 antibody and reduction of cyst burden cannot be directly demonstrated because cyst burden is only inferred before treatment, based on MAG1 antibody level. Direct measurement of brain cysts requires that mice be euthanized. However, we found, as expected from our previous studies^12^, a positive correlation between MAG1 antibody level and the number of brain cysts at the termination of the experiment across both αPD-L1 and isotype control groups (r = 0.69, *p* = 0.012, Fig. 6c). These findings provide indirect support for a dynamic relationship between MAG1 antibody levels and cyst burden.

## Discussion

In mice with chronic *Toxoplasma* infection, we examined the effects of PD-L1 blockade on parasite control, the rescue of exhausted antigen-specific CD8 + T cells, and reversal of neuroinflammation. Consistent with our previous study^23^, this immunotherapy significantly reduced the number of brain tissue cysts. Although the frequency of antigen-specific CD8 + T cells was not affected by PD-L1 blockade, the CD8 + cells produced more IFN-γ. There was a marked increase in serum levels of cytokines and chemokines in αPD-L1-treated mice. While systemic immune responses were enhanced, CNS neuroinflammation was reduced, as evidenced by attenuation of meningeal inflammatory responses, reactive astrocytes, and C1q expression. The reduced inflammatory response is associated with decreased parasite burden. Our results suggest that the inflammatory response, as a consequence of persistent parasite burden, can be reversed, at least partially, by diminishing the parasite burden. Neuroinflammation is a prime component of virtually all neurodegenerative diseases^36^. However, anti-inflammatory and immunosuppressive therapies have performed poorly in clinical trials^37^. Our study provides a proof of concept for the resolution of neuroinflammation via PD-L1 blockade in models of brain infection.

Although PD-L1 blockade did not increase the frequency of IFN-γ–producing antigen-specific CD8 + T cells in the spleen, it augmented IFN-γ production within these cells. IFN-γ is an essential cytokine required for an effective anti-*Toxoplasma* immune response, and CD8 + T cells are a primary source of this cytokine^35^. Previous study showed that the PD-L1 blockade rescued exhausted CD8 + T cells in *Toxoplasma*-infected mice, evidenced by augmentation of IFN-γ production and cytotoxicity^24^. Here, we showed the enhanced capacity of antigen-specific CD8 + T cells to produce IFN-γ, which contributes to improved parasite control. Pathogen-specific T cell polyfunctionality, including IFN-γ-secretion, has been correlated with T cell efficacy, immune protection, and pathogen clearance^38–40^.

Consistent with our previous study^23^, we observed a marked increase in levels of multiple serum cytokines and chemokines in αPD-L1-treated mice. Elevation of serum pro- and anti-inflammatory cytokines, such as IFN-γ, IL-1β, IL-2, IL-4, IL-10, IL-16, and TNF-α, might be a hallmark of the functional cure of *Toxoplasma*-infected mice. Elevation of many different types of chemokines (CCL5, CXCL11, CCL27, CX3CL1, CCL20, and CCL22) demonstrated that PD-L1 blockade activates cross-talk between T cells, macrophages, and dendritic cells. A primary distinction between exhausted and functional CD8 + T cells is the ability to produce cytokines such as IFN-γ, TNF-α, and IL-2 upon T cell receptor stimulation^41^. These biomarkers are produced by both immune and non-immune cells that can interact with each other and elicit biological activity. It is tempting to speculate that the coordinated activation of these cells is key to successful anti-*Toxoplasma* responses.

*Toxoplasma* infection is known to cause an enlargement of the spleen exhibited as an increase in weight and size^33,34^. We observed a further increase in spleen weight in αPD-L1-treated mice with chronic *Toxoplasma* infection, likely attributed to a systemic immune cell expansion. Monoclonal antibodies targeting PD-1 have been shown to have high accumulation in the spleen of mice^42,43^. In mice bearing B16 melanoma tumors, the blockade of PD-1 significantly increased spleen weight^44^. In non-human primates, dual blockade of PD-1 and CTLA-4 showed T cell expansion in the spleen, accompanied by increased spleen weights^45^. We previously reported increased CD3 + T lymphocytes in the spleen of aPD-L1-treated mice, mainly in periarteriolar lymphoid sheaths^23^. It has been suggested that the weight of the spleen can predict the cellular immune response in tumor-bearing mice^46^. In the current study, there was a negative correlation between the number of tissue cysts and spleen weight in aPD-L1- and isotype-treated mice, suggesting parasite clearance is linked to systemic immune responses.

Our results showed reduced CNS neuroinflammation in αPD-L1-treated mice, as evidenced by the decreased inflammatory infiltrates (CD3, CD14) in the meningeal compartment and reduced expression of markers for astrocytes (GFAP) and complement activation (C1q) in the brain. The reason, presumably, is due to the reduced parasite burden failing to trigger a robust inflammatory response. Indeed, the reduction in inflammation-related proteins is correlated with reduced parasite burden. Previous reports using animal models of multiple sclerosis shows meningeal inflammation paralleling remittances and relapses; infiltrates and inflammatory mediators decrease during remission^13,20^. As an important barrier and a gateway into the CNS, inflammation in meningeal compartments could affect CNS inflammation^47^. We previously observed a rapid mobilization of leukocytes into brains via CSF-filled compartments following the PD-L1 blockade^23^. Timing differences after completion of the treatment between the current and our previous study (2 vs. 6 weeks) may account for the distinct findings in meningeal compartments. Parasite clearance can occur rapidly through peripheral immune cell infiltration, but the neuroinflammation triggered by tissue cysts may require time to diminish.

We have previously shown that MAG1 antibodies are a serological marker of cyst burden in the brain^12^. Consistent with the clearance of brain tissue cysts, MAG1 antibody levels declined in mice that received the αPD-L1. Moreover, the number of tissue cysts detected at the end of the experiment correlated with MAG1 antibody levels measured post-treatment. The results suggest that MAG1 antibodies might serve as a biomarker for monitoring the efficacy of anti-*Toxoplasma* agents directed at chronic infection. Moreover, the correlation between MAG1 antibodies and the expression of some inflammation-related proteins suggests that MAG1 antibodies could indicate neuroinflammation triggered by *Toxoplasma*’s brain-dwelling.

Currently, available anti-*Toxoplasma* drugs are ineffective for the clearance of tissue cysts. Employing animal models of *Toxoplasma* infection, we demonstrated that PD-L1 blockade is an effective immune strategy for tissue cysts. This treatment is independent of the infecting strain (virulent vs. avirulent) or host (outbred vs. inbred mice). The treatment exhibited short-term (2 wks) and longer-term (6 wks) efficacy. The findings may have implications in preventing *Toxoplasma*-associated diseases in multiple settings, such as encephalitis and pneumonitis in immunosuppressed patients, congenital neurologic and ocular disease, and acquired ocular disease in immunocompetent persons.

There is an urgent need to develop therapeutic compounds that effectively attenuate neuroinflammation. Unfortunately, compounds that were beneficial in preclinical models of neurological disease, including anti-inflammatory therapies, have largely failed in clinical use^48,49^. Employing animal models of AD and Tau, studies have found that blockade of the PD-1/PD-L1 pathway modified brain pathology and restored cognitive performance^50,51^. These studies suggest systemic immunity should be boosted, rather than suppressed, to drive an immune-dependent cascade needed for brain repair. Our results agree with these studies, suggesting parasite clearance by promoting systemic immunity alleviates neuroinflammation.

## Supplementary Information

Supplementary Information 1.

Supplementary Information 2.

## Data Availability

All data generated or analyzed during this study are included in this published article and its supplementary information files.
