# Supplementary figures and images for: Immunotherapy targeting the PD-1 pathway alleviates neuroinflammation caused by chronic Toxoplasma infection
Source: Sci Rep. 2023 Jan 23;13:1288. doi: 10.1038/s41598-023-28322-8 (PMC9870997; doi:10.1038/s41598-023-28322-8)

The full, uncropped blot images for Fig. 5


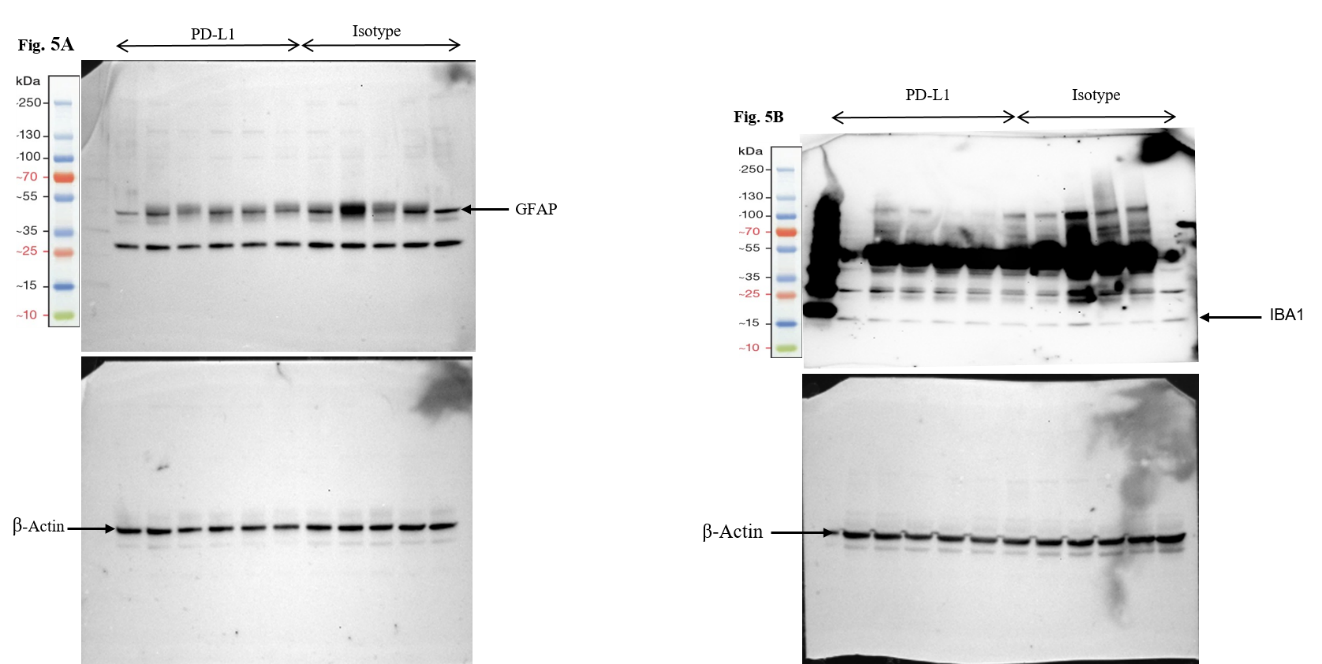

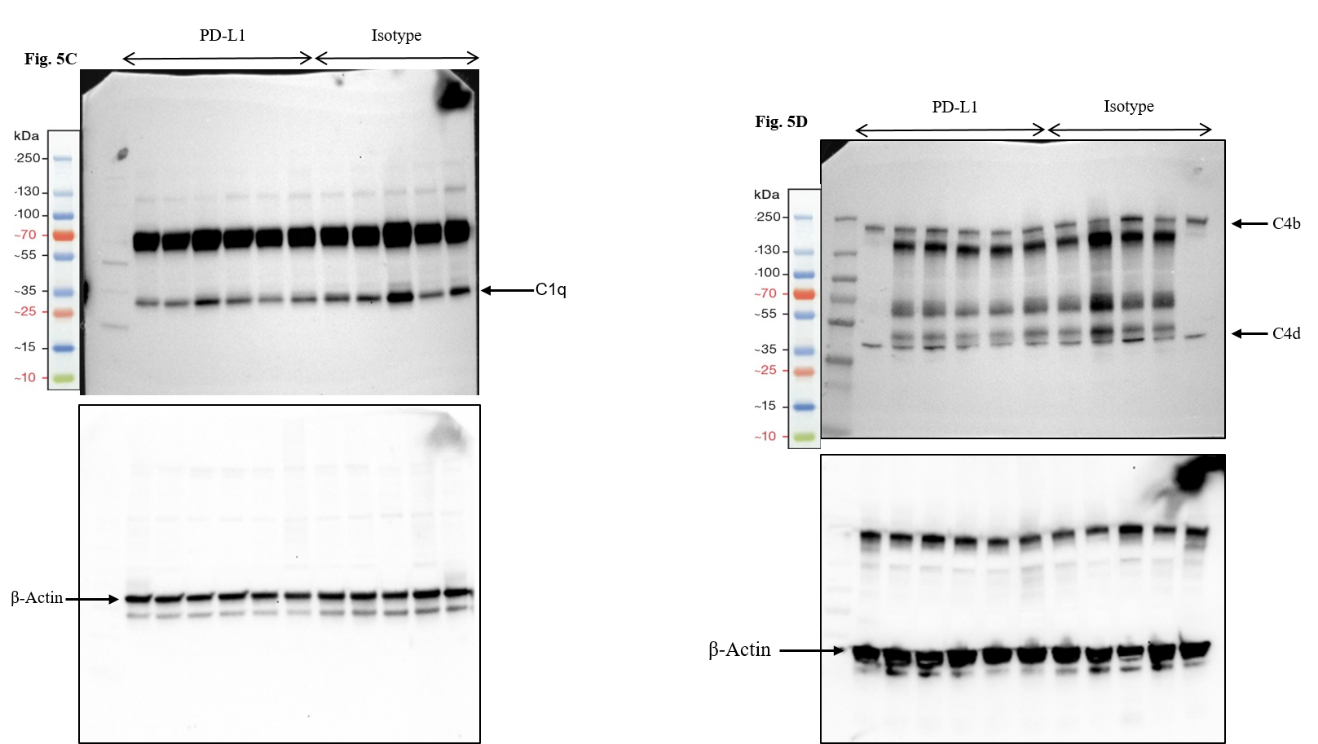

Supplement: Supplementary file 1 — Supplementary Information 1. [file 41598_2023_28322_MOESM1_ESM.docx]
